# The history of African trypanosomiasis

**DOI:** 10.1186/1756-3305-1-3

**Published:** 2008-02-12

**Authors:** Dietmar Steverding

**Affiliations:** 1 BioMedical Research Centre, School of Medicine, Health Policy and Practice, University of East Anglia, Norwich NR4 7TJ, UK

## Abstract

The prehistory of African trypanosomiasis indicates that the disease may have been an important selective factor in the evolution of hominids. Ancient history and medieval history reveal that African trypanosomiasis affected the lives of people living in sub-Saharan African at all times. Modern history of African trypanosomiasis revolves around the identification of the causative agents and the mode of transmission of the infection, and the development of drugs for treatment and methods for control of the disease. From the recent history of sleeping sickness we can learn that the disease can be controlled but probably not be eradicated. Current history of human African trypanosomiasis has shown that the production of anti-sleeping sickness drugs is not always guaranteed, and therefore, new, better and cheaper drugs are urgently required.

## Background

African trypanosomiasis is an infectious disease of humans and animals of similar aetiology and epidemiology. The causative agents of the disease are protozoan parasites of the genus *Trypanosoma *that live and multiply extracellularly in blood and tissue fluids of their mammalian hosts and are transmitted by the bite of infected tsetse flies (*Glossina *sp.). The distribution of trypanosomaisis in Africa corresponds to the range of tsetse flies and comprises currently an area of 8 million km^2 ^between 14 degrees North and 20 degrees South latitude [1]. Throughout history, African trypanosomiasis has severely repressed the economic and cultural development of Central Africa.

African animal trypanosomiasis or nagana disease is caused by *T. congolense*, *T. vivax *and *T. brucei *spp. In wild animals, these parasites cause relatively mild infections while in domestic animals they cause a severe, often fatal disease. All domestic animals can be affected by nagana and the symptoms are fever, listlessness, emaciation, hair loss, discharge from the eyes, oedema, anaemia, and paralysis. As the illness progresses the animals weaken more and more and eventually become unfit for work, hence the name of the disease "N'gana" which is a Zulu word that means "powerless/useless" [2]. Because of nagana, stock farming is very difficult within the tsetse belt.

Human African trypanosomiasis or sleeping sickness is caused by two subspecies of *T. brucei*, *T. brucei gambiense *and *T. brucei rhodesiense*, while the third subspecies, *T. brucei brucei*, is only infectious to animals. *T. b. gambiense *is responsible for the chronic form of sleeping sickness in West and Central Africa, whereas *T. b. rhodesiense *gives rise to the acute form of the disease in East and Southern Africa. There are two distinct stages during the course of sleeping sickness. The first or early stage of the disease, also known as the haemolymphatic phase, is defined by the restriction of the trypanosomes to the blood and lymph system [3]. The symptoms of this stage are fever, headaches, joint pains and itching. The second or late stage of the disease, also known as the neurological phase, is characterised by the presence of the parasites in the cerebrospinal fluid [3]. In general, this is when the typical signs of the disease occur: confusion, disturbed sleep pattern, sensory disturbances, extreme lethargy, poor condition and coma. If left untreated, sleeping sickness patients die within months when infected with *T. b. rhodesiense *or within years when infected with *T. b. gambiense*. Wild and domestic animals may play a major role as parasite reservoirs for human infections with trypanosomes [3-5].

## Prehistory

Phylogenetic reconstruction based on the genes coding for the small subunit ribosomal RNA suggested that all Salivarian trypanosomes (to which African trypanosomes belong) separated from other trypanosomes approximately 300 million years ago [6]. Probably soon after their emergence, Salivarian trypanosomes became gut parasites or commensals of early insects, which evolved around 380 million years ago. With the appearance of tsetse flies some 35 million years ago, trypanosomes have been transmitted to mammals by these bloodsucking insects. The long coexistence of both tsetse flies and game animals may explain why most African wildlife species are tolerant of trypanosomiasis: they become infected by the parasite but show no ill effects [7]. In contrast, domestic animals have yet been unable to develop tolerance or resistance to trypanosome infections within the 13000 years of their breeding.

It is likely that trypanosomiasis has played an important role in early hominid evolution. Probably, the disease had an important role in the selection of trypanosome-resistant early terrestrial hominids. This is evident from the observation that arboreal primates are susceptible to trypanosomiasis while humans, with the exception of *T. b. gambiense *and *T. b. rhodesiense *infections, are resistant [7]. The fact that humans are resistant to all other African trypanosome species indicates that human African trypanosomiasis is a recent event in human development. Presumably the sustained transmission of trypanosomes between tsetse flies and humans in West Africa has led to the evolution of the less virulent *T. b. gambiense *subspecies [7]. In contrast, the *T. b. rhodesiense *subspecies has remained ill-adapted to humans and is transmitted from game animals to humans [7]. The infectivity of *T. b. rhodesiense *to humans is due to a serum-resistant-associated (*SRA*) gene [8]. It seems that the SRA gene originated in a single event and then spread through *T. brucei *in East Africa by genetic exchange [9].

## Antiquity

**Figure 1:**
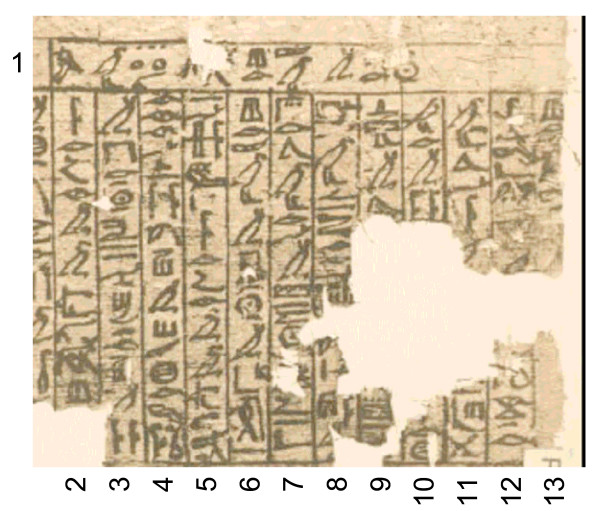
**Last section of the damaged Veterinary Papyrus of the Kahun Papyri about the cattle disease *ushau *dating from 2^nd ^millennium BC**. The translation reads as follows: (1) Title: Treatment of the eyes (?) of a bull with *ushau *in winter. (2) If you see a bull with *ushau *(3) in winter, and he is blinded (?), (4) his two eyes are thick; gash thou as (5) above. If you see a bull (6) with *ushau *in winter from cold, (7) since its arrival in (?) the summer, (8) his temples are wrinkled (?), his eyes are running, his stomach groaning (?), (9) he does not walk (?) .........(10) ............... (11) ............... (12) thou all his body with ......... as is done to one (13) with a bruise (?) [11].

It is well established that in ancient times the north coast of the African continent held more lush vegetation than today [10]. Also the flora and fauna of the Nile valley during the Old Kingdom (3000 BC – 2000 BC) was quite different and probably similar to the current region of the Gazelle River (one of the major tributaries of the Nile River) in Sudan [2]. The distribution of tsetse flies should therefore have extended much more northwards and ranged into the Nile delta. Hence, it is reasonable to assume that shepherds and livestock breeders in these regions experienced the problem of trypanosomiasis. This is also evident from the fact that the Egyptians of the Old Kingdom kept their cattle together with game animals [2]. The ancient Egyptians did this not because they were inexperienced in breeding but could only successfully rear trypanotolerant animals. Further evidence for the presence of trypanosomiasis in ancient Egypt comes from the Veterinary Papyrus of the Kahun Papyri dating from 2^nd ^millennium BC [11] in which a cattle disease is described that resembles nagana (Fig. 1). It seems that an ointment made from the fat of particular birds was used as treatment against the bite of flies [12]. During the course of the Middle Kingdom (2000 BC – 1300 BC) the stream course of the Nile River was adjusted and thus the breeding sites of tsetse flies were largely destroyed. At that time the Egyptians gave up raising game animals and discontinued growing pure breeds of the trypanotolerant aurochs (*Bos primigenius*). Instead, they interbred the aurochs with the more efficient Indian zebu cattle (*Bos indicus*) [2]. The gradual eradication of the tsetse fly due to the progressing regulation of the Nile River eventually allowed the ancient Egyptians to raise pure breeds of zebu cattle [2]. In addition, it is told that the horse, which was not introduced until the 16^th ^century BC, was also difficult to breed in ancient Egypt [2]. It is probable that failure in horse breeding was also due to trypanosome infections by tsetse flies.

## Middle Ages

There are only a few written reports giving evidence for the occurrence of trypanosomiasis in Africa during the Middle Ages. Most of these reports are from the Arabs who kept close trade relations with the West African kingdoms such as Benin, Ghana, Mali and Songhai. One of the first historical records on human trypanosomiasis is by the famous Arabian geographer Abu Abdallah Yaqut (1179–1229). During his journey into Africa he found in the "Country of Gold" (Wangara [13]) an underground village whose inhabitants and even their dogs were just skin and bones and asleep [2]. This scene is reminiscent of the devastating sleeping sickness epidemic in Uganda at the turn of the 20^th ^century. A first case report of sleeping sickness comes from the Arabian historian Ibn Khaldun (1332–1406). In his historical work he reported that a fellow countryman told him about the death of the Sultan Mari Jata, Emperor of Mali, who died of an illness which, according to the description, corresponds to human trypanosomiasis [14]: "He told me that Jata had been smitten by the sleeping illness, a disease which frequently afflicts the inhabitants of that climate, especially the chieftains who are habitually affected by sleep. Those afflicted are virtually never awake or alert. The sickness harms the patient and continues until he perishes. He said that the illness persisted in Jata's humour for a duration of two years after which he died in the year 775 AM (= 1373/74 in our calendar)."

A striking example of the impact of trypanosomiasis on the life and destiny of people is the eastward migration of the Fulbe (Fulani) in the northern parts of West Africa [2]. It is believed that in ancient times the Fulbe people have moved from Egypt or Ethiopia to the area of present-day Senegal. At the beginning of the 13^th ^century when the Sahara was getting increasingly dryer, they were forced to relocate southwesterly into the savannah area. As cattle-breeding was their economic basis, the Fulbe did not risk continuing their migration southward otherwise they would have entered the tsetse belt and lost their herds. Hence, they moved eastward and settled south of the Sahara but north of the tsetse belt in regions with sufficient grazing land.

## Modern Times

### Early Modern Times

In early Modern Times, the history of human African trypanosomiasis is closely linked to the slave trade. First accounts of sleeping sickness came from ship doctors and medical officers who worked for slave-trade companies. As sleeping sickness caused increasing losses, ship-owners and slave-traders pressed their ship doctors to investigate this eerie disease. In 1734, the English naval surgeon John Aktins (1685–1757) published the first accurate medical report on African sleeping sickness [15]. However, whereas Aktins described only the neurological symptoms of the late stage of sleeping sickness, the English physician Thomas Winterbottom (1766–1859) published in 1803 a report referring to the characteristic sign of swollen lymph glands along the back of the neck in the early stage of the disease [15]. He also mentioned that this symptom was known long ago by Arabian slave-traders who refrained from buying slaves with this sign [2]. Although throughout the 19th century, reports on sleeping sickness increased and human African trypanosomiasis became a well-recognised disease, no one had any real idea about the nature of the illness [15].

### Discovery of the tsetse fly-trypanosome complex

**Figure 2:**
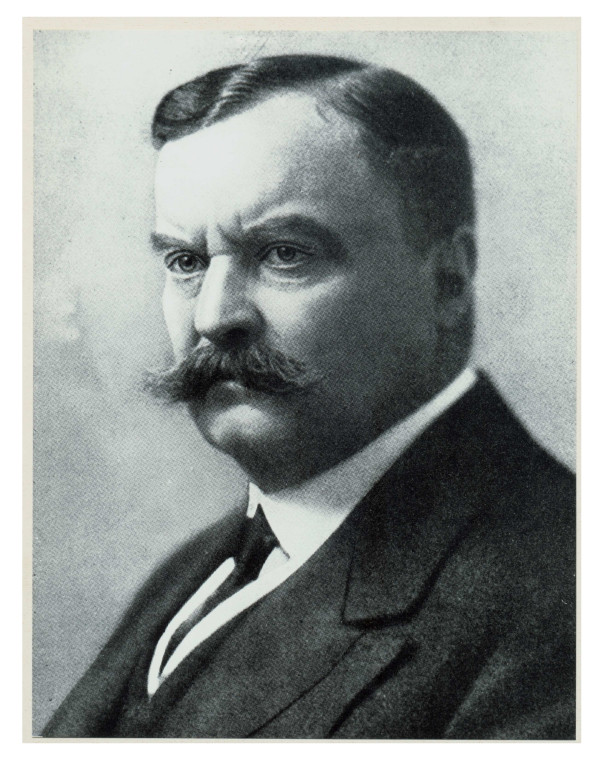
**Sir David Bruce (1855–1931)**. The Scottish bacteriologist identified *T. brucei *as the aetiological agent of nagana disease.

It was the Scottish missionary and explorer David Livingston (1813–1875) who first suggested that nagana is caused by the bite of tsetse flies. In 1852, he reported the occurrence of a disease in the valleys of the Limpopo and Zambezi rivers as well as at the banks of the lakes Nyasa and Tanganyika from which all the cattle he carried died after they have been bitten by tsetse flies [2]. However, it took another 40–50 years until trypanosomes were identified as the causative agents of nagana and sleeping sickness. In 1895, the Scottish pathologist and microbiologist David Bruce (1855–1931) (Fig. 2) discovered *T. brucei *as the cause of cattle trypanosomiasis (cattle nagana) [16]. The first unequivocal observation of trypanosomes in human blood was made by the British Colonial surgeon Robert Michael Forde (1861–1948) in 1901 when he examined a steamboat captain in The Gambia [17]. He first thought that the organisms he found were worms [15] but the English physician Joseph Everett Dutton (1874–1905) identified them as trypanosomes a few months later and proposed in 1902 the species name *Trypanosoma gambiense *(now *T. b. gambiense*) [18]. In the same year, the Italian physician and pathologist Aldo Castellani (1878–1971) found trypanosomes in the cerebrospinal fluid of sleeping sickness patients and suggested that they cause sleeping sickness [15,19]. One year later, Bruce provided conclusive evidence that sleeping sickness is transmitted by tsetse flies [15,20]. At that time, however, he believed that trypanosomes were transmitted mechanically by tsetse flies [15]. It was the German military surgeon Friedrich Karl Kleine (1869–1951) who showed in 1909 the cyclical transmission of *T. brucei *in tsetse flies [21]. This prompted Bruce to change his original opinion of mechanical transmission of trypanosomes, and instead describe the full developmental cycle of the parasites within their insect host [15]. In the meantime, the two other animal pathogenic trypanosome species *T. congolense *and *T. vivax *were discovered in 1904 and 1905 by the Belgian physician Alphonse Broden (1875–1929) [22] and the German naval doctor Hans Ziemann (1865–1905) [23], respectively. The second human pathogenic trypanosome species, *T. rhodesiense *(now *T. b. rhodesiense*), was eventually recovered in 1910 by the parasitologists John William Watson Stephens (1865–1946) and Harold Benjamin Fantham (1876–1937) [24].

### Epidemics and control of the disease

**Figure 3:**
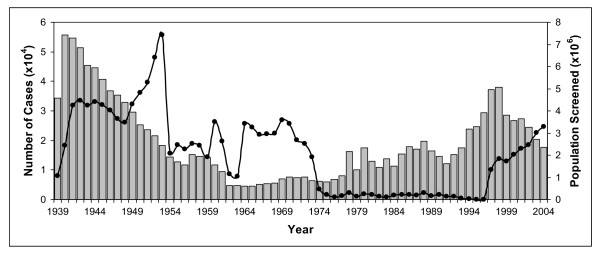
**Number of reported cases of sleeping sickness and population screened, 1939–2004**. Grey columns, number of reported cases; black circles, population screened. Data derived from [27,33].

In the 20^th ^century, Africa saw three severe sleeping sickness epidemics. The first one began in 1896 and lasted until 1906, and affected mainly Uganda and Congo [3]. It was a devastating epidemic with 300,000 and 500,000 people estimated to have died in the Congo Basin and the Busoga focus in Uganda and Kenya, respectively [20,25]. The disastrous effects of the epidemic worried the colonial administrations to such an extent that they sent out scientific missions to investigate the disease (see above) and to develop a cure [2,20]. The French physician Charles Louis Alphonse Laveran (1845–1922) and the French biologist Félix Mesnil (1868–1938) were the first to report in 1902 that sodium arsenite was effective in infected laboratory animals [15]. In 1904, a paper was published by the Canadian doctor Harold Wolferstan Thomas (1875–1931), and the Austrian doctor and zoologist Anton Breinl (1880–1944), informing that the arsenical drug atoxyl could cure experimentally infected animals [2]. It was thought to be better than any other arsenical compound tested so far and relatively atoxic (hence the name) [2]. However, the German physician Robert Koch (1843–1910), who investigated the trypanocidal activity of atoxyl on sleeping sickness patients on the Ssese Islands located in the northwest of Lake Victoria, found that the drug was by no means nontoxic; of 1622 atoxyl-treated patients Koch observed 22 cases of atrophy of the optic nerve with complete blindness [2]. In 1907, Koch informed the German scientist Paul Ehrlich (1854–1915) about these complications and advised him to improve the drug atoxyl [2]. Already in 1904 Ehrlich had become interested in the chemotherapy of trypanosomiasis and had developed the dye trypan red, which proved to be both curative and prophylactic for *T. equinum *(a species of trypanosomes that causes Mal de Caderas in horses in Central and South America) in mice but not for *T. brucei *[26]. Eventually, it was Ehrlich's former assistant Wilhelm Roehl (1881–1929) who in 1916, with the help of a small team of chemists and the German chemical and pharmaceutical company Bayer, developed the first effective drug for treatment of sleeping sickness. The compound, Bayer 205, (later named suramin) is still in use in the therapy of early-stage *T. b. rhodesiense *infections [2]. A year earlier, the American chemist Walter A. Jacobs (1883–1967) and the American immunologist Michael Heidelberger (1888–1991) discovered the organo-arsenical tryparsamide. This was the first drug to treat late-stage sleeping sickness alone, or in combination with suramin, and was also employed in the treatment of animal trypanosomiasis [26]. Both drugs helped to fight the second major sleeping sickness epidemic which began in a number of African countries in 1920 and died down by the late 1940s (Fig. 3) [3,27]. Another important measure towards the control of the 1920s sleeping sickness epidemic was the introduction of mobile teams [20]. This method of systematic case detection and treatment with the aim of elimination of the parasite reservoir was suggested by the French military surgeon Eugène Jamot (1879–1937). In 1926, after long opposition by his superiors in Paris, Jamot was allowed to set up a special service in Cameroon that showed the effectiveness of his approach; within 11 years the prevalence levels of sleeping sickness declined from 60% in 1919 to 0.2–4.1% in 1930 [20]. Subsequently, other colonial powers introduced the method of mobile teams for *T. b. gambiense *sleeping sickness control [20]. Other approaches to the control of African trypanosomiasis were vector control, host reservoir control and game destruction [20]. Vector control was already introduced in 1910 and included the use of differently designed traps and bush clearing. Between 1920 and 1940, reservoir host control and game destruction, which was practised mainly in East Africa on the recommendation of Bruce, resulted in a significant reduction, but never in the extermination, of the tsetse fly population [20]. A third drug for treatment of the early stage of *T. b. gambiense *sleeping sickness, pentamidine, was developed by the English chemist Arthur James Ewins (1882–1958) of the pharmaceutical company May and Baker in 1937 [28]. With the discovery of its insecticidal properties in 1939, DDT was used by 1949 in the hope of freeing large parts of endemic areas from tsetse flies [2,20]. Also in 1949, the arsenical melarsoprol, which was developed by the Swiss pathologist, microbiologist and chemist Ernst Friedheim (1899–1989), was introduced for the treatment of late stage human African trypanosomiasis. It was the first and is still the only effective drug for late stage *T. b. rhodesiense *sleeping sickness. Since the 1950s, several drugs have become available for chemotherapy of animal trypanosomiasis. These include the phenanthridine derivatives homidium bromide (Ethidium^®^, Novidium^®^) and isometamidium chloride (Samorin^®^, Trypamidium^®^), the aminoquinaldine derivative quinapyramine (Anthrycid^®^) and the aromatic diamidine diminazene aceturate (Berenil^®^) [29]. Eventually, the combined employment of chemotherapy, systematic case detection and vector control led to a dramatic reduction in the incidence of sleeping sickness at the beginning of the 1960s (Fig. 3) [27].

Other factors that affected the epidemiology of sleeping sickness in the first half of the last century are the socio-economic conditions created during the colonisation of Africa. An excellent example of this is the sleeping sickness epidemic in the north-central Uele district of the former Belgian Congo, now known as the Democratic Republic of Congo [30]. Colonisation of this region in the first decade of the 19^th ^century was protracted and brutal. Large numbers of people were displaced and many of them experienced famine. This created an ideal environment for spreading the disease and sleeping sickness became increasingly entrenched and epidemic in this region over the next 15 years. It was not until the mid 1920s that medical services were introduced in the Uele district by the colonial powers. Five years later, the epidemic was under control due, as claimed by the Belgian authorities, to the medical interventions. However, improvements in nutrition and hygiene are likely to have had as much to do with the decline of sleeping sickness as the medical interventions [30]. By the 1930s, many people in Belgian Congo no longer suffered from intense social and economic disruption and learned how to better cope with the rules and controls of the colonial powers while the Belgians at the same time ceased their social engineering practice of abrupt resiting of whole communities [30].

By the mid 1960s, most of the trypanosomiasis-endemic countries became independent and were no longer supported by their former colonial powers. In the aftermath of decolonisation, many African countries experienced political instability and economic ruin with a disastrous effect on the health services. After a decade of low endemicity, the control of trypanosomiasis was no longer a priority. As a consequence, control programmes were stopped and population screening declined to very small numbers of people (Fig. 3) [27]. Concerns about the environmental effect of DDT led to a ban of the insecticide in disease vector control worldwide in the 1970s. The result of all this was that since the mid 1970s there has been a steady increase in the number of reported sleeping sickness cases (Fig. 3) [27]. This was the beginning of the third and most recent sleeping sickness epidemic in the 20^th ^century, mainly affecting Angola, Congo, Southern Sudan and the West Nile district of Uganda [3,20]. The situation remained unchanged until 1990 when eflornithine (DL-α-difluoromethylornithine, DFMO), a selective inhibitor of ornithine decarboxylase, was introduced for treatment of late stage *T. b. gambiense *sleeping sickness. Eflornithine was initially developed by scientists at the Merrell Research Institute in Strasbourg for treatment of cancer [31], but was then found to be an effective therapeutic agent against *T. b. gambiense*. Although the administration regime is strict and difficult, eflornithine was a welcome alternative to melarsoprol treatment as it is less toxic.

### Recent developments and current situation

At the turn of the millennium, the scale of sleeping sickness had almost reached, yet again, the levels of the epidemics seen at the beginning of the century (Fig. 3) [27,32]. The situation got even worse as the production of eflornithine was ceased and that of melarsoprol was threatened to be discontinued. Fortunately, in 2001 the World Health Organization (WHO) reached an agreement with the pharmaceutical companies Aventis (now Sanofi-Aventis) and Bayer AG to provide sleeping sickness drugs free of charge for endemic countries [3,32]. The aid organisation Médicins Sans Frontières was commissioned with the distribution of the drugs. By 1997, surveillance had been reinforced and since 1998 the number of new cases has dropped steadily (Fig. 3) [33]. At present, the estimated number of infected patients is thought to be between 50,000 and 70,000 [3].

In 2001, the Organisation of African Unity (OAU) launched a new initiative, the Pan African Tsetse and Trypanosomiasis Eradication Campaign (PATTEC) to eliminate the tsetse fly from Africa [34]. It was planned to employ an area-wide approach using odour-baited traps, insecticide-treated targets and pour-ons and ultra-low volume aerial spraying of insecticides to reduce the tsetse fly population, and finally the sterile male technique to ensure total elimination of the target *Glossina *species [34]. The sterile male technique was successfully used in the eradication of tsetse flies and thus trypanosomiasis on the island of Zanzibar in 1997 [35]. However, in contrast to the Zanzibar project, which worked because it was on an island (isolated area of 1,651 km^2^) infested with only one tsetse fly species, the PATTEC initiative has to deal with a vast area of sub-Saharan Africa (~10 million km^2^) inhabited by at least 7 different *Glossina *species recognised as vectors for transmission of sleeping sickness. Therefore, many scientists are sceptical that the PATTEC project will succeed as similar eradication campaigns failed in the past because the tsetse fly infested areas could not be isolated [36]. The huge costs associated with the eradication project are also a concern as most of the countries involved belong to the most heavily indebted poor countries in the world [36].

The only new drug candidate currently in development for treatment of sleeping sickness is the diamidine pafuramidine (DB289). In January 2007, pafuramidine had completed enrolment for Phase III clinical trials in the Democratic Republic of Congo and Angola [37,38] which is the final step before the compound can be registered as a drug against human African trypanosomiasis. If successful, pafuramidine would be the first orally available treatment for early stage sleeping sickness. Another approach to improve the treatment of sleeping sickness is the development of a combination therapy. Currently, the anti-Chagas disease drug, nifurtimox, is being tested in combination with melarsoprol or eflornithine in a randomized clinical trial in Uganda [39,40].

There is also an urgent need for accurate tools for the diagnosis of human African trypanosomiasis. The existing tests for diagnosis are not sensitive and specific enough, due to the characteristically low number of parasites found in the blood of sleeping sickness patients. Therefore, the Foundation for Innovative New Diagnostics (FIND) and the WHO launched, in 2006, a new initiative for the development of new diagnostic tests to support the control of sleeping sickness [41]. It is expected that the new test will allow for early case detection and simplified staging and, thus, will improve disease management and support for the elimination of sleeping sickness as a public health problem.

## Conclusion

The history of African trypanosomiasis gives an example of how a disease not only affected the evolution of humans but also the cultural and economic development of people in sub-Saharan regions. From the historical events of the 20^th ^century one can learn that a concerted approach of systematic case detection and treatment is the appropriate method for the control of sleeping sickness and that discontinuation of these control measures will lead to re-emergence and spread of the disease. History has also shown that African trypanosomiasis always prevented the introduction of stock farming in endemic areas. A consequence of this is that much of tropical Africa is still present today and has not been converted into grassland for cattle breeding.

## Competing interests

The author declares that he has no competing interests.
